# Robust derivation of epicardium and its differentiated smooth muscle cell progeny from human pluripotent stem cells

**DOI:** 10.1242/dev.119271

**Published:** 2015-04-15

**Authors:** Dharini Iyer, Laure Gambardella, William G. Bernard, Felipe Serrano, Victoria L. Mascetti, Roger A. Pedersen, Amarnath Talasila, Sanjay Sinha

**Affiliations:** Anne McLaren Laboratory for Regenerative Medicine andWellcome Trust-Medical Research Council, Cambridge Stem Cell Institute, University of Cambridge, West Forvie Site, Robinson Way, Cambridge CB2 0SZ, UK

**Keywords:** Human pluripotent stem cells, Epicardium, Vascular smooth muscle cells

## Abstract

The epicardium has emerged as a multipotent cardiovascular progenitor source with therapeutic potential for coronary smooth muscle cell, cardiac fibroblast (CF) and cardiomyocyte regeneration, owing to its fundamental role in heart development and its potential ability to initiate myocardial repair in injured adult tissues. Here, we describe a chemically defined method for generating epicardium and epicardium-derived smooth muscle cells (EPI-SMCs) and CFs from human pluripotent stem cells (HPSCs) through an intermediate lateral plate mesoderm (LM) stage. HPSCs were initially differentiated to LM in the presence of FGF2 and high levels of BMP4. The LM was robustly differentiated to an epicardial lineage by activation of WNT, BMP and retinoic acid signalling pathways. HPSC-derived epicardium displayed enhanced expression of epithelial- and epicardium-specific markers, exhibited morphological features comparable with human foetal epicardial explants and engrafted in the subepicardial space *in vivo*. The *in vitro*-derived epicardial cells underwent an epithelial-to-mesenchymal transition when treated with PDGF-BB and TGFβ1, resulting in vascular SMCs that displayed contractile ability in response to vasoconstrictors. Furthermore, the EPI-SMCs displayed low density lipoprotein uptake and effective lowering of lipoprotein levels upon treatment with statins, similar to primary human coronary artery SMCs. Cumulatively, these findings suggest that HPSC-derived epicardium and EPI-SMCs could serve as important tools for studying human cardiogenesis, and as a platform for vascular disease modelling and drug screening.

## Abstract (precis)

**HIGHLIGHTED ARTICLE**: Epicardial cells and their derivatives are generated from human pluripotent stem cells, an approach that has potential applications in the field of regenerative medicine.

## INTRODUCTION

Epicardium and epicardium-derived cells (EPDCs) play crucial roles in embryonic heart formation. During development, the epicardium primarily contributes to smooth muscle cells (SMCs) and fibroblasts of the coronary vessels (Guadix et al., 2006; Gittenberger-de Groot et al., 1998), to myocardial fibroblasts (Dettman et al., 1998; Manner, 1999), and to a lesser extent to cardiomyocytes (Cai et al., 2008; Zhou et al., 2008a) and coronary endothelial cells (Poelmann et al., 1993; Perez-Pomares et al., 2002a; Katz et al., 2012). The embryonic epicardium also serves as an important signalling centre where reciprocal exchange of paracrine factors between the epicardium and the underlying myocardium promote development of coronary vessels and cardiomyocytes (Wessels and Perez-Pomares, 2004). An emerging paradigm proposes that re-activation of the embryonic developmental program in injured adult epicardial tissues by ectopic signals can mediate epicardium differentiation towards cardiogenic fates or enhance the existing level of signalling activity within the epicardium (Smart et al., 2007). Thus, an *in vitro* model that recapitulates the key events regulating early lineage commitment to epicardium and its derivatives would facilitate efficient and reproducible generation of highly enriched vascular cells for potential applications in vascular disease modelling, drug screening and construction of bioengineered cardiac grafts.

The epicardium develops mostly from the proepicardium, a mesothelial structure in the wall of the pericardial cavity located dorsal to the developing heart tube (Manasek, 1969). The precise origin of the proepicardium is presently unclear. Although some studies in the chick suggest a secondary heart field (SHF) (van Wijk et al., 2009) and lateral plate mesoderm (LM) origin (Bressan et al., 2013), a recent study provides evidence for contribution of somatic mesoderm to proepicardium formation (Schlueter and Brand, 2013). In mice, genetic lineage-tracing studies suggest that the proepicardium originates from NKX2.5- and ISL1-expressing lateral plate/splanchnic mesoderm progenitors (Zhou et al., 2008b). Proepicardial cells migrate onto the outer surface of the heart tube and spread as an epithelial sheet over the rest of the developing heart, thereby forming the epicardium (Manner et al., 2001). Epicardial cells produce a layer of extracellular matrix between the epicardium and the myocardium: the subepicardium. Subsequently, epicardial cells undergo epithelial-to-mesenchymal transition (EMT) and migrate into the subepicardium.

Epicardial EMT is regulated by several signalling molecules, including PDGF (Smith et al., 2011), TGFβ (Bax et al., 2011), FGF (Lavine et al., 2005) and retinoic acid (RA) (von Gise et al., 2011). The epicardium and EPDCs in the subepicardial space are identified by the expression of transcription factors: WT1 (Carmona et al., 2001), TBX18 (Kraus et al., 2001) and TCF21 (Lu et al., 1998). EPDCs migrate into the underlying myocardium where they contribute to coronary vasculature and myocardial cell populations. Signalling pathways that regulate formation of epicardium and EPDCs have been widely studied in avian models (Olivey and Svensson, 2010; Perez-Pomares and de la Pompa, 2011), but are less well defined in mammals and, in particular, in humans.

A recent study by Witty and colleagues (2014) reported the generation of epicardium by differentiating human pluripotent stem cells (HPSCs) to a cardiac fate. Here, we report an alternate method of generating epicardium, epicardium-derived smooth muscle cells (EPI-SMCs) and epicardium-derived cardiac fibroblasts (EPI-CFs) from HPSCs under chemically defined conditions by first inducing an early mesoderm lineage, then LM before further specification to epicardium. We demonstrate that a combination of WNT, BMP and RA signalling promotes robust epicardium differentiation from LM. Our HPSC-derived epicardial cells display characteristic epithelial cell morphology and express elevated levels of epicardial markers (TBX18, WT1 and TCF21), similar to human foetal epicardial outgrowths. Importantly, the epicardial cells undergo EMT and differentiate *in vitro* into mature and functional vascular SMCs (VSMCs), and to some extent into CFs. Moreover, we show that these epicardial cells localise to the subepicardial space of developing chicken embryos and integrate into coronary vessels when injected into the extra-embryonic circulation. Together, these findings demonstrate that HPSCs can be efficiently differentiated to epicardium and its derivatives by recapitulating early developmental events *in vitro*. Functional vascular EPI-SMCs generated using this method will have broad applications in the field of cardiovascular regenerative medicine.

## RESULTS

### Generation of lateral plate/splanchnic mesoderm

**Fig. 1.**
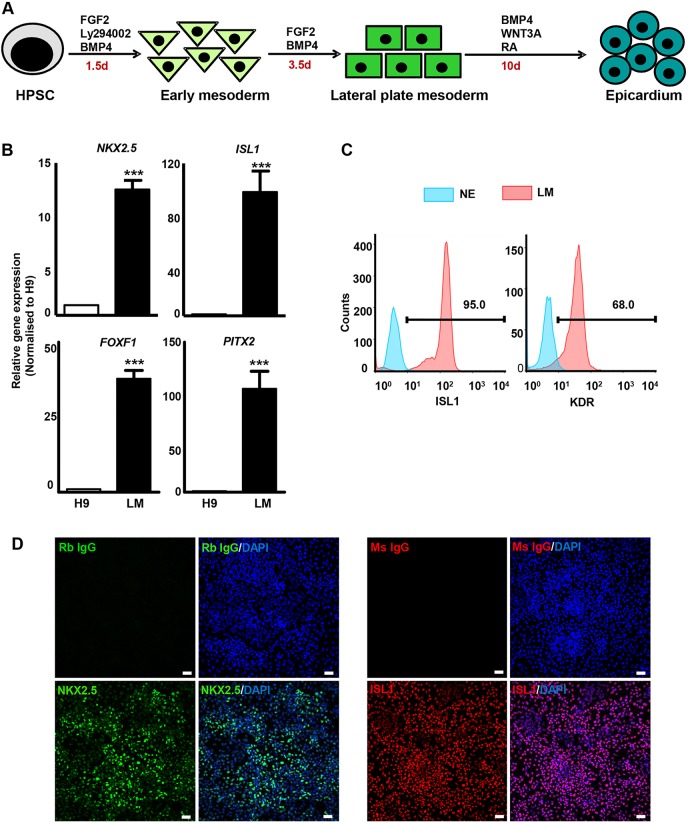
**Induction of lateral plate mesoderm (LM).** (A) Schematic of HPSC differentiation to LM and epicardium. HPSCs were differentiated to early mesoderm using FGF2, Ly294002 and BMP4 for 36 h, and subsequently to LM with FGF2 and BMP4 for 3.5 days. (B) Analysis of LM/splanchnic mesoderm markers in LM by qRT-PCR. Student's *t*-test, ***P<0.001. (C) Percentage of ISL1^+^ and KDR^+^ cells in LM determined by flow cytometry. H9-derived neuroectoderm (NE) was used as a negative control. (D) The majority of LM cells immunostained positive for NKX2.5 and ISL1. Rabbit and mouse IgG isotypes were used as negative controls. Scale bars: 100 μm.

HPSCs (H9 and BHX) were used to generate LM, epicardium and EPI-SMCs. As epicardium develops from LM progenitors, we adopted our previously published protocol to specify LM (Cheung et al., 2012). Briefly, HPSCs were differentiated to early mesoderm using FGF2, Ly294002 and BMP4 for 36 h and subsequently with FGF2 and BMP4 for 3.5 days (Fig. 1A). The generation of LM progenitors from HPSCs mimics the BMP concentration gradient that exists along the primitive streak (PS) (Dosch et al., 1997). We observed a marked increase in the expression of LM markers *NKX2.5* and *ISL1* after 5 days of differentiation (Fig. 1B), as documented previously. The LM cell population also demonstrated enriched expression of *FOXF1* and *PITX2* (Fig. 1B), transcription factors expressed in the LM and splanchnic mesoderm during development (Campione et al., 2001; Mahlapuu et al., 2001). High efficiency of LM specification was observed with over 60% of the derived cells positive for KDR (Fig. 1D), a proximal LM marker (Yamaguchi et al., 1993) that is also expressed by a broad spectrum of mesodermal progenitors that give rise to cardiomyocytes, SMCs and endothelial cells (ECs) (Ema et al., 2006). Over 90% of the cells expressed ISL1 (Fig. 1C), a marker of the SHF (Cai et al., 2003). A vast majority of LM cells also displayed staining for NKX2.5 and ISL1 (Fig. 1D).

### WNT, BMP and RA promote epicardium differentiation

To direct LM to an epicardial lineage, we systematically analysed the effects of various signalling molecules, based on developmental studies in chicken (Kruithof et al., 2006; Schlueter et al., 2006) and zebrafish (Liu and Stainier, 2010). LM was cultured as a monolayer for 10 days under different combinations of growth and regulatory factors. As formation of epicardium occurs at ∼3.5 weeks of human development (1 week after the formation of the first heart field) (Manner et al., 2001), LM cells were initially differentiated for 10 days to identify the best signalling conditions and subsequently for 15 days to optimise the time scale for epicardium differentiation.

**Fig. 2.**
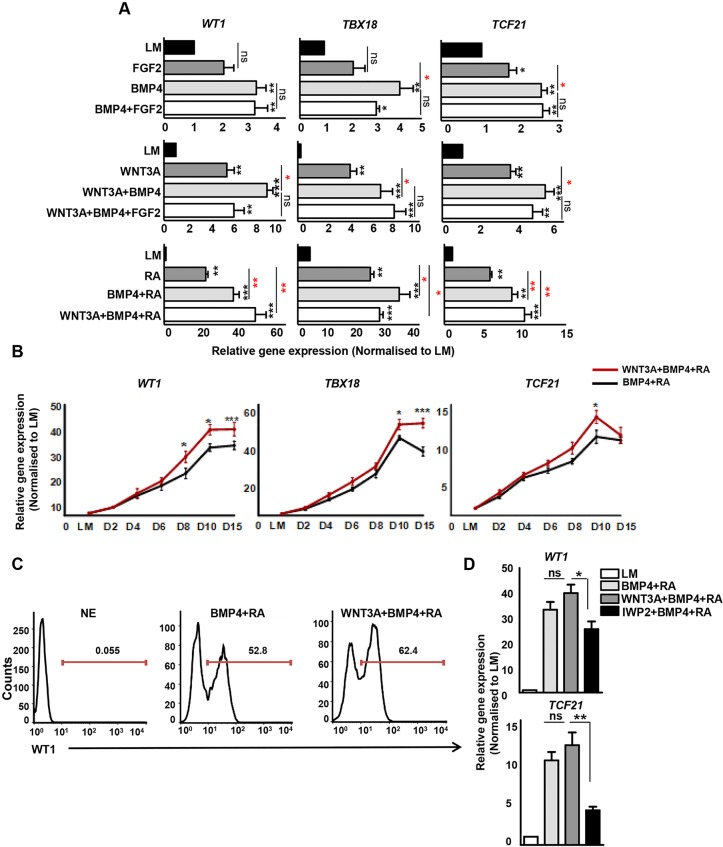
**WNT3A, BMP4 and RA are key regulators of LM differentiation to epicardium.** (A) Expression of epicardial genes by qRT-PCR in epicardium-like cells generated from LM after 10 days of treatment with different combinations of FGF2, BMP4, WNT3A and retinoic acid (RA). Significant differences compared with LM are indicated in black, whereas intergroup differences are indicated in red. ***P<0.001, **P<0.01, *P<0.05. (B) Temporal expression of epicardial genes in BMP4+RA- and WNT3A+BMP4+RA-treated cells determined by qRT-PCR. ***P<0.001, *P<0.05. (C) Percentage of WT1^+^ cells in BMP4+RA and WNT3A+BMP4+RA-treated day 10 epicardial cells. H9-derived neuroectoderm (NE) served as negative control. (D) Inhibition of endogenous WNT signalling by differentiating LM with IWP2 resulted in reduced expression of epicardial genes (*WT1* and *TCF21*). **P<0.01, *P<0.05.

Correctly balanced BMP and FGF signals regulate proepicardium formation in the chick (Kruithof et al., 2006; van Wijk et al., 2009). The requirement for BMP signalling in proepicardium specification has also been reported in zebrafish (Liu and Stainier, 2010). Accordingly, we first tested FGF2, BMP4 or a combination of both on LM and looked for the expression of *WT1*, *TBX18* and *TCF21* by qRT-PCR. BMP4 promoted the expression of epicardial markers in a dose-dependent manner (from 10 ng/ml to 200 ng/ml) (supplementary material Fig. S1A). Higher concentrations of BMP4 (100 ng/ml and 200 ng/ml) had the same effect as that of BMP4 (50 ng/ml) used to generate LM and thus a constant concentration of 50 ng/ml of BMP4 was used throughout the study. FGF2 alone induced a small increase in the expression of *TCF21*, but not *TBX18* and *WT1* (Fig. 2A). To determine optimal FGF2 concentration, LM cells were treated with 50 ng/ml of FGF2 (as employed previously for LM differentiation) and a higher concentration of FGF2 (100 ng/ml). Both showed similar results and failed to significantly induce epicardial gene expression (supplementary material Fig. S1B). Treatment with BMP4 demonstrated increased induction of epicardial markers compared with treatment with FGF2 alone (Fig. 2A). However, the combination of FGF2 with BMP4 failed to promote further expression of epicardial markers suggesting that, in the HPSC system in the presence of BMP4, no additional FGF2 was required to induce epicardium differentiation. This provided a platform for investigating additional signalling pathways involved in epicardium/coronary vessel development.

During development, Wnt/β-catenin signalling promotes mesoderm formation (Takada et al., 1994) and has biphasic effects during cardiogenesis (Naito et al., 2006). Loss-of-function studies in mice lacking Dkk1 and Dkk2 suggest that Wnt signalling plays an important, although not a defined, role in the specification of epicardium during development (Phillips et al., 2011). To investigate the role of WNT/β-catenin signalling, we treated LM cells with WNT3A, resulting in relatively higher epicardial gene expression compared with cells differentiated with BMP4 and FGF2 (Fig. 2A). Titration studies revealed optimal expression of all three epicardial genes (*TBX18*, *WT1* and *TCF21*) with 25 ng/ml of WNT3A (supplementary material Fig. S1A). In the presence of BMP4, WNT3A promoted increased expression of epicardial markers (Fig. 2A). However, treatment with FGF2 in the combined presence of WNT3A and BMP4 did not augment epicardial gene expression any further (Fig. 2A), confirming our initial observations that FGF2 signalling by itself or in combination with other factors was inadequate in driving LM to an epicardial fate. Although BMP4 and WNT3A promoted RNA expression of epicardial markers, they failed to induce substantial expression of WT1 at the protein level when examined by flow cytometry (supplementary material Fig. S2).

We next investigated the effects of RA signalling, an important regulator of epicardial EMT and coronary development in the specification of epicardium. qRT-PCR revealed a substantial increase in the expression of *TBX18*, *WT1* and *TCF21* in RA-treated LM cells compared with other signalling conditions (Fig. 2A). Furthermore, flow cytometry analysis revealed WT1 expression in RA-treated cells in contrast to cells differentiated with WNT3A, BMP4 and FGF2, with more WT1^+^ cells after treatment with 4 μM RA than with 1 μM RA (supplementary material Fig. S2). The highest expression of epicardial markers was noted with 4 μM and 6 μM RA (supplementary material Fig. S1A). However, increased cell death after 3-4 days of differentiation was observed in cells treated with 6 μM RA (data not shown). Therefore, 4 μM RA was determined as the optimal concentration for deriving epicardium-like cells. Interestingly, RA induced greater expression of *TBX18* and *TCF21* with BMP4 than without BMP4 (Fig. 2A). Last, we tested the effects of WNT3A in the combined presence of RA and BMP4. Treatment with WNT3A+BMP4+RA resulted in significantly higher levels of *TBX18*, *WT1* and *TCF21*, similar to BMP4+RA (Fig. 2A). Taken together, these results demonstrate that modulation of WNT3A, BMP4 and RA signalling can lead to differentiation of an epicardium-like cell population from LM under chemically defined conditions.

### Endogenous WNT signalling is sufficient but not optimal for epicardium differentiation

Since the two best conditions for epicardium differentiation appeared to be BMP4+RA and WNT3A+BMP4+RA, we investigated more closely the requirement for WNT signalling. LM cells were differentiated for 15 days using BMP4+RA with and without WNT3A. Both conditions demonstrated an equivalent increase in the expression of epicardial markers at early and middle stages of differentiation, although the presence of WNT3A resulted in a modest increase in epicardial markers at later stages (Fig. 2B). Interestingly, the expression of epicardial genes either peaked or plateaued at day 10. Meanwhile at day 15, we noted higher levels of SMC (*CNN1*) and cardiomyocyte (*TNNT2*) markers compared with the day 10 cell population (supplementary material Fig. S3A), suggesting the onset of EMT with prolonged differentiation. However, at the protein level, day 10 epicardial cells did not express TNNT2 (supplementary material Fig. S3B), whereas a highly specific TNNT2 expression was observed in HESC-derived cardiomyocytes (supplementary material Fig. S3B and Movie 1), generated using a recently reported method (Mendjan et al., 2014). Flow cytometry revealed a higher percentage of WT1^+^ cells using WNT3A+BMP4+RA compared with BMP4+RA (Fig. 2C), suggesting that WNT3A in the combined presence of BMP4 and RA was more effective in directing LM to an epicardial lineage. The WNT3A+BMP4+RA treated cells also demonstrated a significant decrease in pluripotency (*NANOG* and *POU5F1*) and LM (*NKX2.5* and *ISL1*) markers (supplementary material Fig. S3C), providing additional evidence for HPSC and LM differentiation towards an epicardial lineage.

As BMP4+RA and WNT3A+BMP4+RA were comparable in promoting epicardium differentiation until day 10, we investigated the role of WNT signalling in our system by inhibiting endogenous WNT using IWP2. LM cells were differentiated with BMP4+RA+IWP2 for 10 days and compared with cells differentiated with WNT3A+BMP4+RA and BMP4+RA. IWP2-treated cells demonstrated significantly lower levels of *WT1* and *TCF21* than the other two groups, suggesting an important role for endogenous WNT signalling in epicardium specification (Fig. 2D). Together, these results demonstrate that endogenous WNT signalling in the presence of BMP4 and RA is sufficient for directing LM to an epicardial fate. However, administration of WNT3A is beneficial for sustained expression of epicardial markers, in particular at later stages of differentiation. As the presence of WNT3A induced higher gene and protein expression of epicardial markers, we identified treatment with WNT3A+BMP4+RA as the optimum condition for robust differentiation of an epicardium-like cell population from LM. As highest epicardial marker expression was observed at day 10, this was chosen as the ideal time course for epicardium differentiation.

### Characterisation of HPSC-derived epicardial cells

**Fig. 3.**
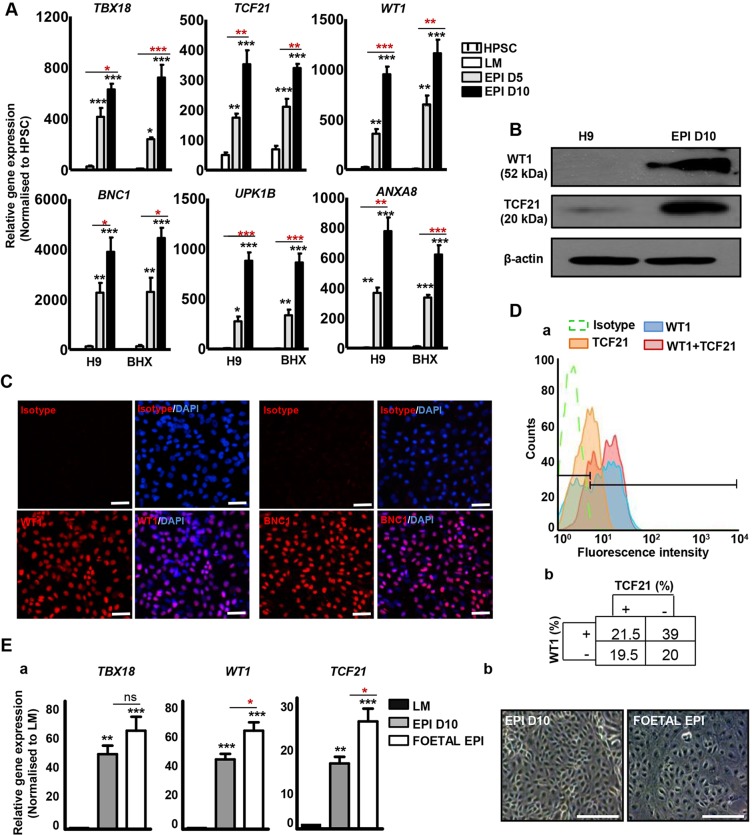
**Characterisation of HPSC-derived epicardium.** (A) Analysis of epicardial markers in H9 and BHX-derived epicardium-like cells by qRT-PCR. Significant differences compared with H9 are indicated in black, whereas intergroup differences are in red. ***P<0.001, **P<0.01, *P<0.05. (B) Western blot showing WT1 and TCF21 expression in H9 and H9-derived day 10 (EPI D10) epicardial cells. β-Actin was used as a loading control. (C) Immunocytochemistry for WT1 and BNC1 in EPI D10. Rabbit IgG isotype used as negative control. Scale bars: 100 μm. (D) (a) Flow cytometry analysis of WT1^+^, TCF21^+^ and WT1^+^/TCF21^+^ cells in EPI D10. Rabbit IgG isotype was used as a negative control. (b) Percentage of cells uniquely expressing WT1, TCF21 and WT1/TCF21, and cells not expressing either of the two markers. (E) (a) Expression of epicardial markers in EPI D10 and human foetal epicardial outgrowths (FOETAL EPI) by qRT-PCR. Significant differences compared with LM are in black, whereas intergroup differences are in red. ***P<0.001, **P<0.01, *P<0.05. (b) Bright-field images of EPI D10 and FOETAL EPI. Scale bars: 100 μm.

To confirm reproducibility of the differentiation process, BHX HiPSCs along with H9 HESCs were differentiated to LM and subsequently to an epicardial lineage with WNT3A+BMP4+RA. Both H9 and BHX-derived epicardial cells displayed high levels of *TBX18*, *WT1* and *TCF21* (Fig. 3A). We also examined the expression of *BNC1*, *UPK1B* and *ANXA8*, genes highly expressed in the adult mouse epicardium (Bochmann et al., 2010). Our HPSC-derived epicardial cells displayed high expression of all six epicardial markers (Fig. 3A) with negligible expression in HPSCs and LM. WT1 (52 kDa) and TCF21 (20 kDa) proteins were also detected in the epicardial cells by western blotting, although not in H9 HESCs (Fig. 3B). H9-derived epicardial cells also demonstrated extensive nuclei-specific staining for WT1 and BNC1 (Fig. 3C), further confirming the expression of these markers at the protein level. We then asked to what extent epicardial markers were co-expressed by the same cells or whether distinct subsets existed within the epicardium with specific marker expression. As all the best-performing antibodies for flow cytometry were raised in rabbit, H9-derived epicardial cells were either singly or co-stained for WT1 and TCF21 using only an anti-rabbit secondary. Flow cytometric analyses revealed 60.5% WT1^+^ cells, 40.7% TCF21^+^ cells and 79.7% WT1^+^ and/or TCF21^+^ cells (Fig. 3Da). From these data, we deduced the following subsets: WT1^+^/TCF21^−^ cells (39%), TCF21^+^/WT1^−^ (19.5%), TCF21^+^/WT1^+^ (21.5%) and WT1^−^/TCF21^−^ (20%). These results provided some insight into heterogeneity in transcription factor expression in human epicardial cells.

The expression levels of *TBX18*, *WT1* and *TCF21* were comparable between H9-derived epicardium and human foetal epicardial outgrowths (albeit slightly higher in foetal in the case of *WT1* and *TCF21*) (Fig. 3Ea). A regular cobblestone epithelial pattern was observed in both cell types (Fig. 3Eb). H9-derived epicardial cells and foetal epicardial outgrowths also displayed comparable nuclear staining for WT1 (supplementary material Fig. S4). To obtain a homogenous epicardial cell population, we flow-sorted the LM for KDR (supplementary material Fig. S5A) and differentiated KDR^+^ cells with WNT3A+BMP4+RA. Epicardial cells generated from the KDR^+^ and unsorted LM were examined for the expression of *TBX18*, *WT1* and *TCF21* by qRT-PCR. Both groups displayed similar expression of all three epicardial markers and no significant differences in gene expression were noted after 5 and 10 days of differentiation (supplementary material Fig. S5B). Very few KDR^−^ cells adhered to the tissue culture plates and hence could not be differentiated. Thus, we concluded that the unsorted LM population gave a robust yield of epicardial cells and obviated any need for cell sorting.

### HESC-derived epicardial cells survive, integrate and differentiate *in vivo*

**Fig. 4.**
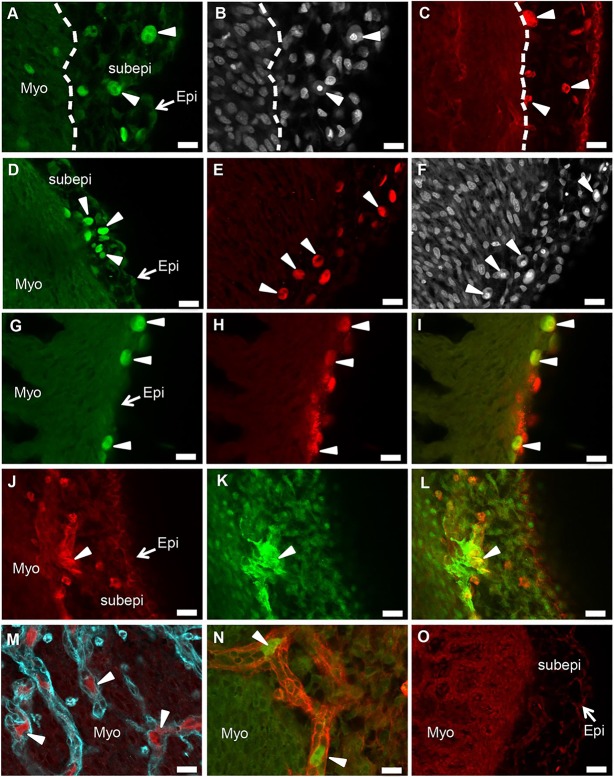
**HESC-derived epicardial cells survive and differentiate *in vivo*.** (A-C) Fluorescent (GFP^+^ and mStrawb^+^) human epicardial cells detected in the subepicardial region at the base of the chicken heart (arrowheads). (B) Human cells in A are identifiable by bright and distinct Hoechst 33342 staining. (D-F) Fluorescent human epicardial cells detected in the subepicardial region in the middle part of the heart (arrowheads). (F) Human cells in E are identifiable by bright and distinct Hoechst 33342 staining. (G,H) Epicardial cells (GFP^+^) that localised at the apex of the heart (G) expressed WT1 (H; indicated by arrowheads). (I) Co-expression of WT1^+^ and GFP^+^ human cells. (J,K) A few engrafted mStrawb^+^ epicardial cells (J) expressed ACTA2 (K), suggesting differentiation to SMCs *in vivo*. (L) Cells co-expressing mStrawb and ACTA2 (indicated by arrowheads). (M,N) GFP^+^ and mStrawb^+^ epicardial cells detected within lectin-stained (in cyan and red) coronary vessels. (O) Subepicardial region in a chicken embryo heart injected with mStrawb^+^ human neural crest cells. Scale bars: 100 μm. Myo, myocardium; Epi, epicardium; Subepi, subepicardium.

Several studies have reported the use of chicken embryos as a host for studying the differentiation potential of human stem cells (Goldstein et al., 2002; Park et al., 2009). We hypothesised that injecting HESC-derived epicardial cells into developing embryos at a stage when the epicardium is formed would promote engraftment in the subepicardial space, eventually contributing to the coronary vasculature. Extra-embryonic intravascular delivery has been previously demonstrated as an efficient method to deliver HESCs to vascularised tissues (Boulland et al., 2010). We therefore administered fluorescent (GFP^+^ or mStrawb^+^) HESC-derived epicardial cells into the extra-embryonic circulation of chicken embryos at Hamburger and Hamilton stage (HH) 24, a developmental stage when the epicardium is completely formed and epicardial cells are migrating into the myocardium (Martinsen, 2005). We harvested the embryos at HH34, when coronary arteries start developing and appear on the surface of the heart. To detect transplanted cells, we used whole-mount confocal immunofluorescence imaging of the heart*.* We detected 20-50 GFP^+^ and mStrawb^+^ epicardial cells per embryo, predominantly in the subepicardial space between the epicardium and myocardium at the base of the heart (Fig. 4A-F) where the subepicardial region was the thickest (supplementary material Fig. S6). Few GFP^+^/mStrawb^+^ were also seen at the apex of the heart (Fig. 4G-I), where the subepicardial/epicardial layer was relatively thinner (supplementary material Fig. S6). HESC-derived epicardial cells could be clearly discriminated from the host cells by their cell size, strong green or red fluorescence (Fig. 4A,C,D,E,G,H,I) and distinct nuclei (detected by Hoechst 33342 staining) (Fig. 4B,F). HESC-derived epicardial cells at the apex of the heart expressed WT1 (Fig. 4G-I), indicating that these cells retain their epicardial cell identity *in vivo*. Some of the engrafted epicardial cells (Fig. 4J,L) also expressed ACTA2 (Fig. 4K,L), suggesting the onset of EMT and SMC differentiation *in vivo*. To identify functional blood vessels, harvested hearts were stained with biotinylated *Sambucus nigra* lectin. Integration of HESC-epicardial cells in lectin-labelled coronary vessels could be distinctly seen (Fig. 4M,N). As negative control, we injected FRSC-derived neural crest cells (F.S. and W.G.B., unpublished) into the extra-embryonic circulation of HH24 embryos and processed the surviving embryos at HH34. We could not detect any human neural crest cells in the subepicardial space (Fig. 4O), implying that HESC-derived epicardial cells uniquely localise to the subepicardial space *in vivo*. Total number of surviving chicken embryos after injection of human epicardial cells, HH stages used for injections and the number of surviving embryos with engrafted human epicardial cells in the subepicardial space and coronary vessels are summarised in Table 1*.* Together, these results suggest that HESC-derived epicardial cells have access to the subepicardial niche *in vivo* and have the potential to contribute to coronary vascular development.

**Table 1.**
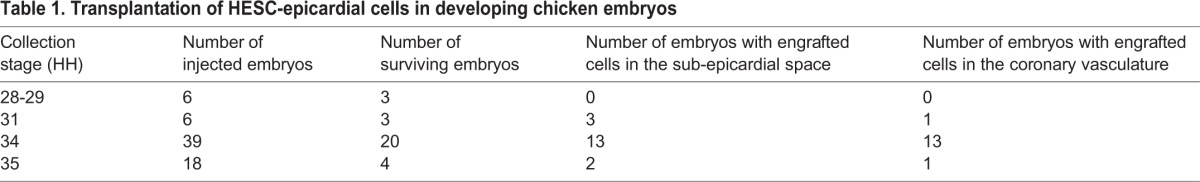
**Transplantation of HESC-epicardial cells in developing chicken embryos**

### Epicardial cells undergo EMT to differentiate into smooth muscle cells and cardiac fibroblasts *in vitro*

**Fig. 5.**
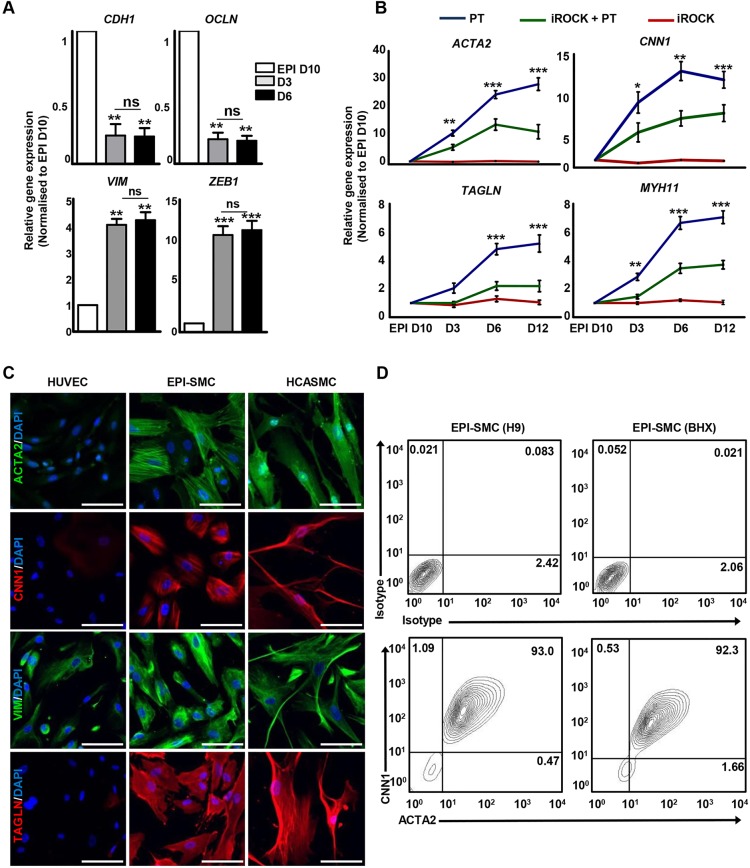
**Epicardium-derived SMC differentiation *in vitro*.** (A) Epithelial and mesenchymal marker expression in day 10 epicardial cells (EPI D10) and epicardium-derived SMCs (EPI-SMCs) after 3 (D3) and 6 (D6) days of differentiation with PDGF-BB and TGF-β1 (PT). ***P<0.001, **P<0.01. (B) SMC marker expression by qRT-PCR in EPI-SMCs differentiated with PT in the presence and absence of p160 Rho-kinase inhibitor (iROCK) after 3, 6 and 12 days of differentiation. Significant differences between PT and iROCK+PT are indicated in black. ***P<0.001, **P<0.01, *P<0.05. (C) EPI-SMCs after 12 days of PT treatment expressed mesenchymal (VIM) and SMC (ACTA2, CNN1 and TAGLN) markers, similar to human coronary artery SMCs (HCASMCs). SMC marker expression was absent in HUVECs. Scale bars: 100 μm. (D) Percentage of ACTA2^+^ and CNN1^+^ cells in H9 and BHX-derived EPI-SMCs. Mouse IgG isotypes served as negative controls.

Once the epicardium is formed, epicardial cells invade the subepicardial space, undergo EMT and largely differentiate into SMCs and cardiac fibroblasts (CFs) (Gittenberger-de Groot et al., 1998; Guadix et al., 2006; Zhou et al., 2008a). Two important regulators of EMT are PDGF (Smith et al., 2011) and TGFβ (Austin et al., 2008), while our group previously reported that mature VSMCs could be obtained from distinct embryological lineages by PDGF-BB and TGFβ1 (PT) treatment (Cheung et al., 2012). We adopted a similar approach to initiate epicardial EMT and promote SMC differentiation. Our H9-derived epicardial cells expressed high levels of epithelial markers (*CDH1* and *OCLN*) that significantly decreased within 3 days of differentiation with PT, while expression of mesenchymal markers (*VIM* and *ZEB1*) was increased (Fig. 5A). Another important mediator of epicardial EMT and coronary SMC differentiation is the RhoA-RhoK signalling pathway (Lu et al., 2001). To determine the role of RhoA in our system, we differentiated epicardial cells with PT in the presence of Y27632 (iROCK), a selective p160 Rho-kinase inhibitor. Inhibition of RhoA signalling (iROCK+PT) resulted in reduced SMC gene expression (*ACTA2*, *CNN1*, *TAGLN* and *MYH11*) compared with EPI-SMCs differentiated using PT (Fig. 5B). The expression of SMC markers increased over time with PT treatment and plateaued between day 6 and day 12 (Fig. 5B). The HPSC-derived EPI-SMCs demonstrated similar gene expression levels to those of the positive control HCASMCs (supplementary material Fig. S7A). In addition to qRT-PCR, the expression of SMC markers in EPI-SMCs was examined using immunocytochemistry, flow cytometry and western blot analysis. Human umbilical vein endothelial cells (HUVECs) served as a negative control. EPI-SMCs homogenously expressed SMC (ACTA2, CNN1 and TAGLN) and mesenchymal (VIM) markers (Fig. 5C). The expression levels of all SMC markers were noticeably similar between EPI-SMCs and HCASMCs, whereas negligible expression was detected in HUVECs (Fig. 5C). Flow cytometry analysis also correlated with the gene expression data. EPI-SMCs after 12 days of PT treatment culminated in >90% ACTA2^+^/CNN1^+^ cells (Fig. 5D). Western blot analysis revealed expression of MYH11 (200 kDa), a mature and discriminating marker of SMCs in our EPI-SMCs (supplementary material Fig. S7B).

**Fig. 6.**
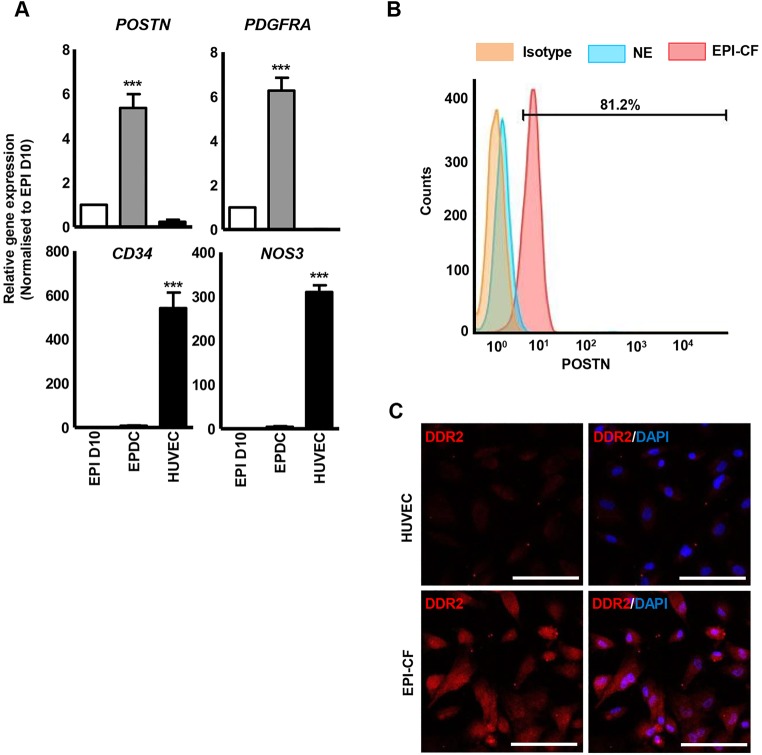
**Epicardium-derived cardiac fibroblast differentiation.** (A) Analysis of fibroblast (*PDGFRA* and *POSTN*) and endothelial (*NOS3* and *PECAM1*) markers in epicardium-derived cells (EPDCs) obtained with VEGF and FGF treatment. ***P<0.001. (B) Percentage of POSTN^+^ cells in epicardium-derived cardiac fibroblasts (EPI-CFs) determined by flow cytometry. Rabbit IgG isotype and HUVECs were used as negative controls. (C) The majority of EPI-CFs immunostained positive for DDR2, whereas HUVECs displayed negligible expression. Scale bars: 100 μm.

While the contribution of epicardium to SMCs and CFs is well established, contribution to ECs and cardiomyocytes is still debatable (Merki et al., 2005; Christoffels et al., 2009). We examined the differentiation potential of HESC-derived epicardium to a CF or EC fate by differentiating the cells with VEGF and FGF for 12 days. The differentiated cells displayed high levels of *POSTN* and *PDGFRA* (Fig. 6A), markers that are robustly expressed in CFs (Snider et al., 2009; Smith et al., 2011). Conversely, these cells displayed negligible expression of endothelial markers (*CD34* and *NOS3*) (Fig. 6A), suggesting that the epicardial cells most likely adopt a CF fate. Flow cytometric analysis further revealed >80% POSTN^+^ cells in the EPI-CFs (Fig. 6B). Similarly, a large proportion of EPI-CFs immunostained positively for DDR2 (Fig. 6C), a marker commonly used to identify CFs (Camelliti et al., 2005). Together, these results demonstrate that HESC-derived epicardial cells recapitulate embryonic developmental events and can differentiate into mature VSMCs or CFs.

### Functional characterisation of epicardium-derived smooth muscle cells

**Fig. 7.**
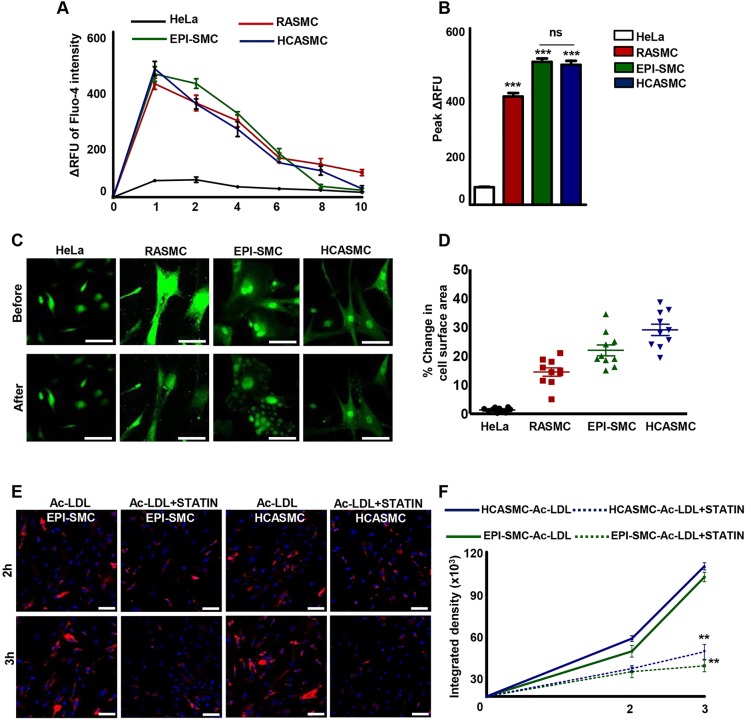
**Functional characterisation of epicardium-derived SMCs.** (A) Change in the relative fluorescence unit (ΔRFU) of Fluo-4 AM-loaded HeLa cells, rat aortic SMCs (RASMC), human coronary artery (HCASMC) and epicardium-derived SMCs (EPI-SMC) by flow cytometry over 10 min after the addition of carbachol. (B) Peak ΔRFU 1 min after carbachol addition. Differences in Fluo-4 intensity compared with HeLa cells. ***P<0.001. (C) Fluo-4 AM-loaded cells displayed a change in cell surface area following carbachol stimulation. (D) EPI-SMCs, RASMCs and HCASMCs displayed 10-30% decreases in cell surface area with negligible change in HeLa cells. (E) Uptake of Alexa Fluor 594-conjugated acetylated low density lipoprotein (Ac-LDL) in EPI-SMCs and HCASMCs appears as red droplets after 2 and 3 h of incubation. Nuclei counterstained with DAPI (blue). Effective lowering of LDL uptake observed after treatment with atorvastatin (Ac-LDL+statin) in both cell types. (F) Quantification of Alexa Fluor 594 intensity in the absence (solid line) and presence (dashed line) of atorvastatin. **P<0.01. Scale bars: 100 μm.

The principal function of mature VSMCs is to regulate blood flow through the vasculature by contraction and relaxation. Calcium (Ca^2+^) channels participate in the regulation of cytoplasmic calcium, which regulates smooth muscle contraction (Webb, 2003). Physiological agonists stimulate influx of extracellular calcium and/or release of stored calcium by opening a variety of channels in the plasmalemma or sarcoplasmic reticulum (Berridge, 1997). Agonist-induced Ca^2+^ fluxes have been previously reported in murine (Sinha et al., 2006) and human (Cheung et al., 2012) ESC-derived SMCs. We investigated the functional ability of EPI-SMCs by preloading the cells with a calcium-sensitive dye Fluo-4 AM and stimulating them with the cholinergic receptor agonist, carbachol. EPI-SMCs showed an increase in Fluo-4 intensity by flow cytometry within a minute of carbachol treatment (green line, Fig. 7A**)**, indicating an increase in intracellular calcium signalling. After 2 and 4 min of stimulation, Fluo-4 intensity decreased, reaching basal levels by 6 min. A similar trend was observed in HCASMCs (blue line) and RASMCs (red line) but not in HeLa cells (black line, negative control). The peak fluorescence response of EPI-SMCs, HCASMCs and RASMCs after 1 min of carbachol stimulation is illustrated in Fig. 7B. Contraction was assessed by measuring the change in cell surface area before and after carbachol treatment. Ten cells were randomly chosen within a field of view for calculating the change in cell surface area before and after carbachol addition. Between a 10 and 15% decrease was observed in low-passage RASMCs while over 20% reduction was observed in EPI-SMCs and HCASMCs (Fig. 6C,D). HeLa cells, used as a negative control, did not show any change in cell surface area.

HiPSCs have been employed in a limited number of studies to model VSMC diseases such as Hutchinson Gilford Progeria (Liu et al., 2011; Zhang et al., 2011a) and Williams Syndrome (Ge et al., 2012). However, in contrast to these rare syndromic disorders, very few common multifactorial vascular diseases such as atherosclerosis have been modelled using HPSCs. A key step in atherosclerotic plaque development is the uptake of modified low density lipoprotein (LDL) by VSMC- and macrophage-expressed scavenger receptors. This accumulation of lipid in vessel wall cells leads to the formation of foam cells and is a key driver of the inflammatory response in atherosclerosis. Three-hydroxy-3-methyl glutaryl co-enzyme A (HMG-CoA) reductase inhibitors, also known as statins, are widely used therapeutic agents in atherosclerosis and act by reducing circulating LDL levels through effects on hepatic LDL clearance. Statins are also thought to have direct effects on vessel wall cells independent of LDL levels in part by inhibiting scavenger receptor expression and thus limiting the generation of foam cells (Hofnagel et al., 2006). As proof of concept, we tested the ability of our EPI-SMCs to model this key patho-physiological step in atherogenesis and their response to statins.

To assess LDL uptake and effect of statins, EPI-SMCs and HCASMCs were incubated with Alexa Fluor 594 conjugated to acetylated LDL (Ac-LDL) for 2 and 3 h respectively. Uptake of Ac-LDL appeared within the cytoplasm of EPI-SMCs and HCASMCs as bright-red droplets (Fig. 7E) with increased uptake after 3 h of incubation compared with 2 h. When EPI-SMCs and HCASMCs were pre-treated with atorvastatin for 45 min, there was a pronounced decrease in Ac-LDL uptake within 2-3 h of atorvastatin treatment (Fig. 7E,F). Together, these results confirmed that our EPI-SMCs were functional and could have potential implications for vascular disease modelling and drug testing.

## DISCUSSION

In this study, we report a simple chemically defined monolayer method to generate epicardium and its differentiated SMC and CF progeny from the LM. Specification of LM from HPSCs by mimicking BMP gradient along the PS has been recently reported by our group (Cheung et al., 2012). As the epicardial lineage originates from lateral plate/splanchnic mesoderm, we extended this approach by patterning the LM to an epicardial fate using a combination of cytokines inferred from developmental insights. We demonstrate that HPSC-derived epicardial cells resemble human foetal epicardial outgrowths in morphology and gene expression. Importantly, the HPSC-epicardial cells showed *in vivo* functionality by homing to the subepicardial niche and contributing to the coronary vasculature in developing chick embryos. These *in vitro* generated epicardial cells also undergo EMT to form SMCs and we show proof of concept data that suggest these SMCs have potential for drug-testing applications. In addition to VSMCs, we demonstrate that HPSC-epicardial cells have the potential to differentiate into CFs.

Our findings reveal a requirement for RA, BMP and WNT signalling in specifying an epicardium-like lineage from LM. More specifically, we show that RA signalling is absolutely necessary for the generation of epicardium, as epicardial marker expression fails to occur in its absence. The importance of RA signalling in early stages of heart development is supported by studies in mice where embryos lacking RA receptor RXRα developed SHF defects (Ryckebusch et al., 2008) and absence of Raldh2 resulted in delayed epicardium formation (Jenkins et al., 2005). Several studies have discussed the role of RA in promoting epicardial EMT, myocardial proliferation and cardiac morphogenesis. Whether or not RA signalling is directly involved in proepicardium/epicardium development *in vivo* is still unclear. Consistent with previous reports (Braitsch et al., 2012), we show that RA plays a major role in inducing the expression of WT1 and TCF21 in HPSC-epicardial cells. We speculate that the need for exogenous RA may be due to lack of specific Raldh2-producing inducers in the LM/LM-derived cells, insufficient endogenous RA synthesis or species-specific requirement for RA in human epicardium development. As epicardium-derived RA signalling promotes myocardial growth (Pérez-Pomares et al., 2002b) and RA treatment has previously been shown to promote atrial cell identity (Xavier-Neto et al., 1999; Zhang et al., 2011b), it is possible that some myocardial cells are induced by RA treatment in our cultures, as evidenced by *TNNT2* expression at prolonged periods of differentiation. Further investigation of subsets within the heterogeneous HESC-epicardium may provide insight into the number and type of myocardial cells produced.

*Ex vivo* studies on proepicardial explant cultures of chicken embryos have suggested an important role for FGF2 signalling in proepicardium development (Kruithof et al., 2006). Interestingly, in our system, FGF2 signalling failed to induce the expression of epicardial genes. One possible explanation for this differential requirement of FGF2 signalling is the different stages of development employed to study epicardial differentiation in the two systems. It is also possible that FGF signalling maintains cell proliferation and growth within the proepicardium and does not influence proepicardial/epicardial cell identity (Torlopp et al., 2010). Other possibilities include species-specific (avian versus mammalian) variation in FGF requirement and differences between *in vitro* and *in vivo* conditions.

In addition to RA signalling, we demonstrate the requirement for BMP and WNT signalling pathways in promoting epicardium differentiation. A recent study by Witty et al. also identified BMP and WNT signalling as important regulators for HPSC-derived epicardium specification (Witty et al., 2014). Witty and colleagues found that whereas low levels of endogenous BMP signalling were required for cardiomyocyte development from cardiogenic mesoderm, increased signalling through exogenously added BMP resulted in epicardial specification. Low levels of WNT activity were also required for epicardium development. Interestingly, high WNT activation could replace the need for exogenous BMP but there was no evidence of synergy and indeed the combination of exogenous BMP and high WNT activation reduced the expression of WT1. In our study we have examined a greater range of epicardial markers, TBX18 and TCF21, in addition to WT1, and also found that BMP and WNT signalling were required to induce epicardium. In contrast to Witty and colleagues, we found that the effects of BMP4 and WNT were additive. This difference may relate to the fact that we used recombinant WNT rather than a GSK3β small molecule inhibitor that might have nonspecific effects at higher concentrations. Our results also demonstrate that, in the continued presence of BMP4 and RA signalling, endogenous WNT signalling is sufficient to drive LM to an epicardium-like cell fate. However, exogenous activation of the WNT/β-catenin pathway supports sustained expression of epicardial genes. Furthermore, in our system, WNT and BMP alone induced epicardial gene expression but generated relatively low levels of epicardial proteins in the absence of RA. It is difficult to compare these findings with those of Witty et al. as they did not quantify expression of epicardium-specific protein, such as WT1, except by limited immunofluorescence studies, and the proportion of their cells expressing epicardial markers was consequently unclear. By contrast, our results provide some insight into the transcriptional heterogeneity within human epicardial cultures. The proportion of WT1^+^/TCF21^+^ in HESC-epicardium was comparable with the subset of WT1^+^/TCF21^+^ cells identified in the chick and mouse epicardium (Braitsch et al., 2012). Identification of other subsets, e.g. TBX18^+^/WT1^+^, TBX18^+^/TCF21^+^ and SCX^+^/SEMA3D^+^ may further facilitate the identification of molecularly distinguishable cell populations with distinct differentiation potential, as previously reported (Katz et al., 2012).

We have used monolayer differentiation throughout to generate epicardium and EPI-SMCs, which differs from the combined embryoid body and monolayer differentiation approach used by Witty and colleagues (2014). Furthermore, it is currently unclear whether the proepicardium originates from the posterior region of the SHF or directly from the adjacent splanchnic mesoderm (Zhou et al., 2008b; Mommersteeg et al., 2010). As there is increasing evidence that the proepicardium comprises a heterogeneous population of cells, then using an unpatterned LM as in our study has both practical and theoretical advantages for generating the full range of proepicardial/epicardial cells (Bollini et al., 2014). To summarise, our approach of generating an epicardium-like cell population from HPSC is based on similar principles reported by Witty et al. but varies in the starting cell population and emphasis on WNT and RA signalling.

We addressed the authenticity of our HESC-epicardium. First, it displayed close similarities to human foetal epicardial outgrowths in morphology and gene expression levels of epicardial markers. Second, when injected into stage-matched chicken embryos, our HESC-epicardial cells localised to the subepicardial space, contributing to developing coronary vasculature and forming SMC-like cells. Finally, similar to embryonic epicardium cells, our HPSC-epicardial cells displayed EMT to give rise to VSMCs that were functionally identical to HCASMCs both in terms of physiological, pathological and therapeutic responses. Besides, EPDC differentiation to SMCs and CFs, and not to ECs supports findings from *in vivo* studies (Cai et al., 2008; Zhou et al., 2008a). We therefore conclude that the present protocol constitutes a valuable and robust platform for epicardial differentiation into potentially clinically useful cells for therapies, disease modelling and drug development.

The ability to efficiently generate epicardium and EPI-SMCs by recapitulating early developmental events could serve as an important tool with which to study the mechanisms of human cardiogenesis and provide insights into why atherosclerosis particularly affects SMCs lining the coronary arteries. Dissecting the molecular basis of epicardium and EPDC specification would not only help to understand developmental processes and mechanisms of cardiovascular diseases but would also facilitate stem cell-based regenerative therapies to regenerate and repair injured cardiac tissues. Human iPSC-derived epicardial cells and their derivatives could also serve as a useful tool to study high-risk regions in the human genome, for example the 9p21 variant that influences risk of coronary artery disease (Samani et al., 2008). The ability to model certain aspects of atherosclerosis, such as uptake of LDL and its subsequent lowering by atorvastatin administration, makes our system an excellent platform for modelling vascular diseases and screening drugs. Bringing together the insights gained from developmental biology and the huge potential of HPSCs in generating large numbers of epicardium and EPI-SMCs could pave the way for significant advancements in stem cell based cardiac regeneration.

## MATERIALS AND METHODS

### HPSC culture

Human embryonic stem cells (HESCs; H9 line, Wicell, Madison,WI) and the human induced pluripotent stem cell (hIPSC) line BHX (Cambridge Biomedical Research Centre hIPSC Core Facility), collectively termed HPSCs, were cultured under chemically defined conditions as described previously (Brons et al., 2007). Briefly, HPSCs were cultured in a chemically defined medium (CDM) containing bovine serum albumin fraction A (CDM-BSA) supplemented with Activin A (10 ng/ml, R&D systems) and FGF2 (12 ng/ml, R&D systems) on gelatine-coated plates. CDM-BSA comprised Iscove's modified Dulbecco's medium (Gibco) plus Ham's F12 NUT-MIX (Gibco) medium in a 1:1 ratio, supplemented with Glutamax-I, BSA (5 mg/ml; Europa Bioproducts), chemically defined lipid concentrate (Life Technologies), transferrin (15 µg/ml, Roche Diagnostics), insulin (7 µg/ml, Roche Diagnostics) and monothioglycerol (450 μM, Sigma).

### HPSC differentiation

Differentiation to specific embryonic lineages was performed in CDM-PVA, which has the same composition as CDM-BSA, with polyvinyl alcohol (PVA, 1 mg/ml, Sigma) instead of BSA. For early mesoderm differentiation, cells were grown in CDM-PVA with FGF2 (20 ng/ml), LY294002 (10 μM, Sigma) and BMP4 (10 ng/ml, R&D) for 36 h then treated with CDM-PVA with FGF2 (20 ng/ml) and BMP4 (50 ng/ml) for 3.5 days to generate LM as previously described (Cheung et al., 2012). To generate epicardium, LM cells were differentiated in CDM-PVA with BMP4 (50 ng/ml), recombinant human WNT3A (25 ng/ml, R&D systems) and RA (4 μM, Sigma) at a seeding density of 2.5×10^3^/cm^2^ for 10 days. To investigate the role of WNT pathway, LM cells were differentiated with IWP2 (2 μM, Tocris) along with BMP4 and RA. Subsequently, epicardial cells were re-suspended in CDM-PVA with PDGF-BB (10 ng/ml, Peprotech) and TGFβ1 (2 ng/ml, Peprotech), designated as PT for 12 days. Epicardial cells were differentiated with PT in the presence of Y27632 (2 μg/ml, Calbiochem) to study the role of RhoA/RhoK in EPI-SMC differentiation. To generate EPI-CFs, epicardial cells were differentiated in CDM-PVA with VEGF (50 ng/ml, Peprotech) and FGF2 (50 ng/ml) for 12 days. HESCs were grown in CDM-PVA with FGF2 (12 ng/ml) and SB431542 (10 μM, Tocris) for 7 days to generate neuroectoderm (Vallier et al., 2009).

### Primary human foetal epicardial culture

Human foetal tissues were obtained following therapeutic pregnancy interruption performed at Cambridge University Hospitals NHS Foundation Trust with ethical approval (East of England Research Ethics Committee) and informed consent in all instances. To induce epicardial outgrowths, 7- to 10-week-old human foetal hearts were cultured in a 1:1 mixture of Dulbecco's modified Eagle's medium (DMEM, Sigma) and Medium 199 (M199, Sigma) containing 100 U/ml penicillin, 100 μg/ml streptomycin and 10% heat inactivated foetal bovine serum (FBS, Sigma). Hearts were placed in a gelatine-coated culture dish (Corning) capped with a glass coverslip (VWR). After 3-4 days of culture, when epithelial outgrowths were visible, the coverslips and the remaining tissue pieces were removed. Epicardial outgrowths were then either fixed with 4% paraformaldehyde (PFA, Affymetrix) for immunocytochemistry or used for RNA extraction.

### Fluorescent HESC lines

H9 ESCs were transfected with a lentiviral vector (LV-indLS1) containing a fluorescent luciferase strawberry reporter (Rodriguez et al., 2014) using Lipofectamine 2000 (Invitrogen). Fluorescent red ESC clones (FRSC) were selected and expanded. We also used a H9-derived HESC line that constitutively expressed GFP (a kind gift from Dr Ludovic Vallier, Stem Cell Institute, Cambridge, UK) generated using the pTP6 vector as previously reported (Teo et al., 2011).

### Quantitative real-time polymerase chain reaction

Total RNA was extracted using the RNeasy mini kit (Qiagen). cDNA was synthesised from 250 ng RNA using the Maxima First Strand cDNA Synthesis kit (Fermentas). Quantitative real-time polymerase chain reaction (qRT-PCR) reaction mixtures were prepared with SYBR green PCR master mix (Applied Biosystems) and run on the 7500 Fast Real-time PCR system. C_T_ values were normalised to porphobilinogen deaminase (PBGD). Primer sequences are listed in supplementary material Table S1.

### Immunocytochemistry

Adherent cells were fixed using 4% PFA, permeabilised with 0.5% Triton-X100 (Sigma) and blocked with 3% BSA (Sigma)/PBS for 60 min at room temperature. Primary antibody incubations were performed at 4°C overnight and Alexa Fluor-tagged secondary antibody (Invitrogen) was applied for 45 min at room temperature. Nuclei were counterstained with DAPI (10 μg/ml, Sigma). Images were acquired on a Zeiss LSM 700 confocal microscope and analysed with ImageJ software. For basonuclin staining, cells were fixed with acetone:methanol (1:1). Epitopes were blocked with normal horse serum (Vector labs) and all endogenous biotin, biotin receptors and avidin-binding sites were blocked with the avidin/biotin blocking kit (Vector labs). Cells were incubated with basonuclin antibody (in 5% BSA and 0.1% Triton-X100) overnight and incubated with horse anti-rabbit IgG conjugated to biotin (Vector labs) for 1 h at room temperature. Streptavidin-conjugated Alexa Fluor 488 (Invitrogen) was used to visualise labelled cells and DAPI was used to counterstain the nuclei. Antibodies used for immunocytochemical analysis are listed in supplementary material Table S2.

### Flow cytometry

Cells were incubated with Cytofix/Cytoperm Fixation solution (BD Biosciences) for 20 min at 4°C, then washed with Perm Wash Buffer/PBS (1×, BD Biosciences) and incubated in primary antibody for 2 h at room temperature. Cells were then incubated in Alexa Fluor-tagged secondary antibody for 1 h at room temperature. Samples were run on a Beckman Coulter CyAN_ADP_ flow cytometer. Datasets were analysed using FlowJo.

### Contraction study

Low-passage rat aortic SMCs (RASMCs), primary human coronary artery SMCs (HCASMCs, Promocell), HeLa cells and EPI-SMCs were preloaded with the calcium-sensitive fluorophore Fluo-4 AM (2.5 μM, Molecular Probes) for 1 h at 37°C. Cells were then trypsinised and treated with carbachol (100 μM, Sigma). Intracellular calcium flux was measured using the FL1 channel of the CyAN ADP flow cytometer. To quantify change in cell surface area, cells were incubated with Fluo-4 AM in normal extracellular solution (NES; 140 mM NaCl, 5 mM KCl, 2 mM CaCl_2_, 1 mM MgCl_2_, 10 mM glucose and 10 mM HEPES, pH 7.3) for 1 h at room temperature. Images were acquired before and after carbachol addition using a Zeiss LSM 700 confocal microscope. Ten cells were randomly picked from a field of view and change in cell surface area of fluorescent cells was assessed using ImageJ software.

### Low density lipoprotein (LDL) uptake assay

EPI-SMCs and HCASMCs were cultured in DMEM-F12 (Gibco) with 10% FBS for 48 h. Cells were serum starved for 12 h then incubated with 100 μM atorvastatin (Sigma) for 45 min at 37°C. Subsequently, cells were incubated with 50 μg/ml of Alexa Fluor 594-conjugated AcLDL (Life Technologies) and 100 μM of atorvastatin for 2 or 3 h. Cells were then fixed and counterstained with DAPI. Images were obtained using a Zeiss LSM confocal microscope. LDL uptake was measured using the ImageJ integrated density measurement tool. Average integrated density was normalised per nucleus.

### Microinjection of HPSC-derived epicardium in chicken embryos

Chicken (*Gallus gallus domesticus*) eggs (Winter Egg Farm, Cambridge, UK) were incubated in a digital cabinet incubator (OVA Easy 380, Brinsea) until Hamburger Hamilton developmental stage 24 (HH24). A small window was made and 500-1000 epicardial cells were administered into the extra-embryonic vessels. HESCs were fully differentiated to epicardial cells before administration into the chicken embryos. H9 (GFP)-derived epicardial cells are referred to as GFP^+^ and FRSC-derived epicardial cells as mStrawb^+^. The window was covered with parafilm (VWR) and eggs were placed horizontally in the incubator until HH34.

### Whole-mount immunofluorescence

Whole chicken embryo hearts were fixed overnight in 4% PFA at 4°C. Hyaluronidase (1 mg/ml; Sigma) with 0.01% Triton-X100 was used for 45 min under rotation at room temperature to remove the cardiac jelly and ensure antibody penetration. The hearts were then blocked in PBS containing 0.8% Triton-X100 (PBT) with 3% milk for at least 2 hours at 4°C under rotation and then incubated with primary antibodies (in PBT/3% milk) overnight at 4°C under constant agitation. Subsequently, hearts were washed for 6 h with PBT at 4°C on a rotating wheel, then incubated overnight with secondary antibodies and Hoechst 33342 (2 μg/ml, Sigma) in PBT/3% milk at 4°C. The hearts were fixed for 1 h at room temperature with 2% PFA and 0.1% glutaraldehyde (Sigma)/PBS, then placed for 1 hour at room temperature in PBS containing 50% glycerol (Sigma) and overnight at 4°C in PBS containing 70% glycerol. The hearts were mounted on microscope cavity slides (Fisher Scientific) using DABCO (Sigma) mounting medium. Images were acquired using a Zeiss LSM 700 confocal microscope.

### Western blot

Samples were lysed in ice-cold RIPA buffer and 10 μg of whole-cell lysate was resolved with SDS-PAGE gel then transferred to polyvinylidine difluoride membranes (Merck Millipore). Membranes were blocked with 5% milk in Tris-buffered saline and 0.05% TWEEN 20 (TBS-T, Sigma) for 1 h at room temperature, followed by primary antibody overnight at 4°C. Anti-rabbit HRP (Sigma) or anti-mouse HRP (Sigma) secondary antibodies were used. Bands were visualised using ECL western blotting detection reagents (Pierce). Primary antibodies used for flow cytometry, immunocytochemistry, whole-mount immunofluorescence are listed in supplementary material Table S2.

### Statistics

One-way ANOVA (Tukey's multiple comparisons test) was used to determine statistically significant differences between the groups unless otherwise mentioned. Results are presented as mean±s.e.m. *P* values of 0.05 or less were considered statistically significant. All experiments represent the results of at least three independent biological replicates.

## Supplementary Material

Supplementary Material
