# Robust derivation of epicardium and its differentiated smooth muscle cell progeny from human pluripotent stem cells

**DOI:** 10.1242/dev.136143

**Published:** 2016-03-01

**Authors:** Dharini Iyer, Laure Gambardella, William G. Bernard, Felipe Serrano, Victoria L. Mascetti, Roger A. Pedersen, Sanjay Sinha, Amarnath Talasila

There were two errors published in Development **142**, 1528-1541.

LM cells were seeded at a density of 2.5×10^4^/cm^2^. To generate EPI-CFs, epicardial cells were differentiated in CDM-PVA with VEGF-B (and FGF2). Although the errors are minor and do not affect the science presented, they are important if others wish to replicate the protocol.

The authors apologise to readers for these mistakes.

