# Supplementary material for: Robust derivation of epicardium and its differentiated smooth muscle cell progeny from human pluripotent stem cells
Source: Development. 2015 Apr 15;142(8):1528–41. doi: 10.1242/dev.119271 (PMC4392600; doi:10.1242/dev.119271)
Supplement: Supplementary Material [file supp_142_8_1528__index.html]

Supplementary Material 

# Robust derivation of epicardium and its differentiated smooth muscle cell progeny from human pluripotent stem cells

## DEV119271 Supplementary Material

**Files in this Data Supplement:**

- Supplementary Material
