# Supplementary material for: Robust derivation of epicardium and its differentiated smooth muscle cell progeny from human pluripotent stem cells
Source: Development. 2015 Apr 15;142(8):1528–41. doi: 10.1242/dev.119271 (PMC4392600; doi:10.1242/dev.119271)
Supplement: Supplementary Material [file supp_dev.119271_DEV119271supp.pdf]

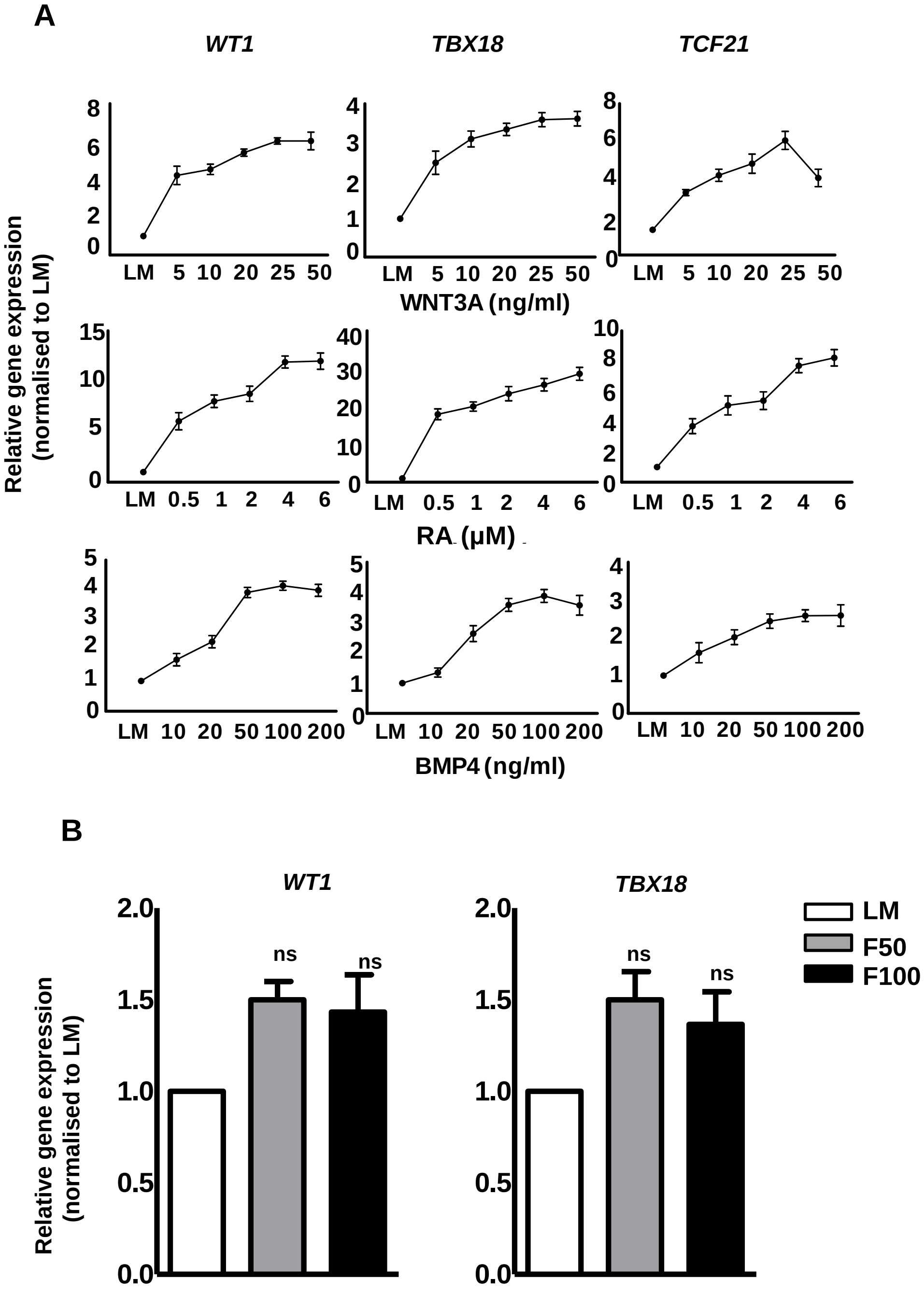

Fig. S1 Titration of WNT3A, BMP4 and RA and their effect on epicardial gene expression. (A) High expression of *TBX18*, *WT1* and *TCF21* was noted with increasing doses of WNT3A. Highest expression of all three epicardial genes was observed at a final concentration of 25 ng/ml. Enhanced epicardial gene expression was noted in cultures supplemented with 4 μM and 6 μM of all trans retinoic acid (RA). Enriched expression of epicardial markers was noted with increasing doses of BMP4 which plateaued at higher concentrations (50, 100 and 200 ng/ml). (B) Analysis of epicardial markers in LM cells differentiated with two different concentrations of FGF2. No significant induction in the expression of *TBX18* and *WT1* was observed.

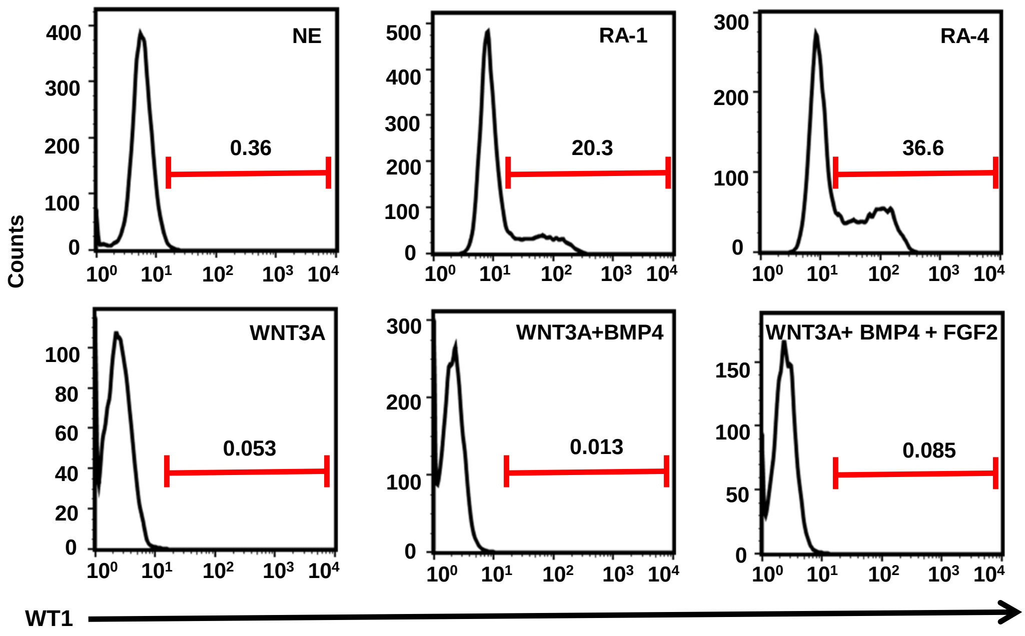

Fig. S2. Flow cytometric analysis of WT1 expression in cells differentiated using WNT3A and RA treated cells. Lateral plate mesoderm cells differentiated with WNT3A, WNT3A+BMP4 and WNT3A+BMP4+FGF2 failed to induce any WT1 expression in the differentiated cells after 10 days of differentiation. Addition of RA promoted expression of WT1 in the differentiated cells. A higher concentration of RA (4 μM) resulted in a larger percentage of $WT1^+$ cells compared to cells differentiated with a lower concentration of RA (1 μM). H9-derived neuroectoderm (NE) cells were used as a negative control.

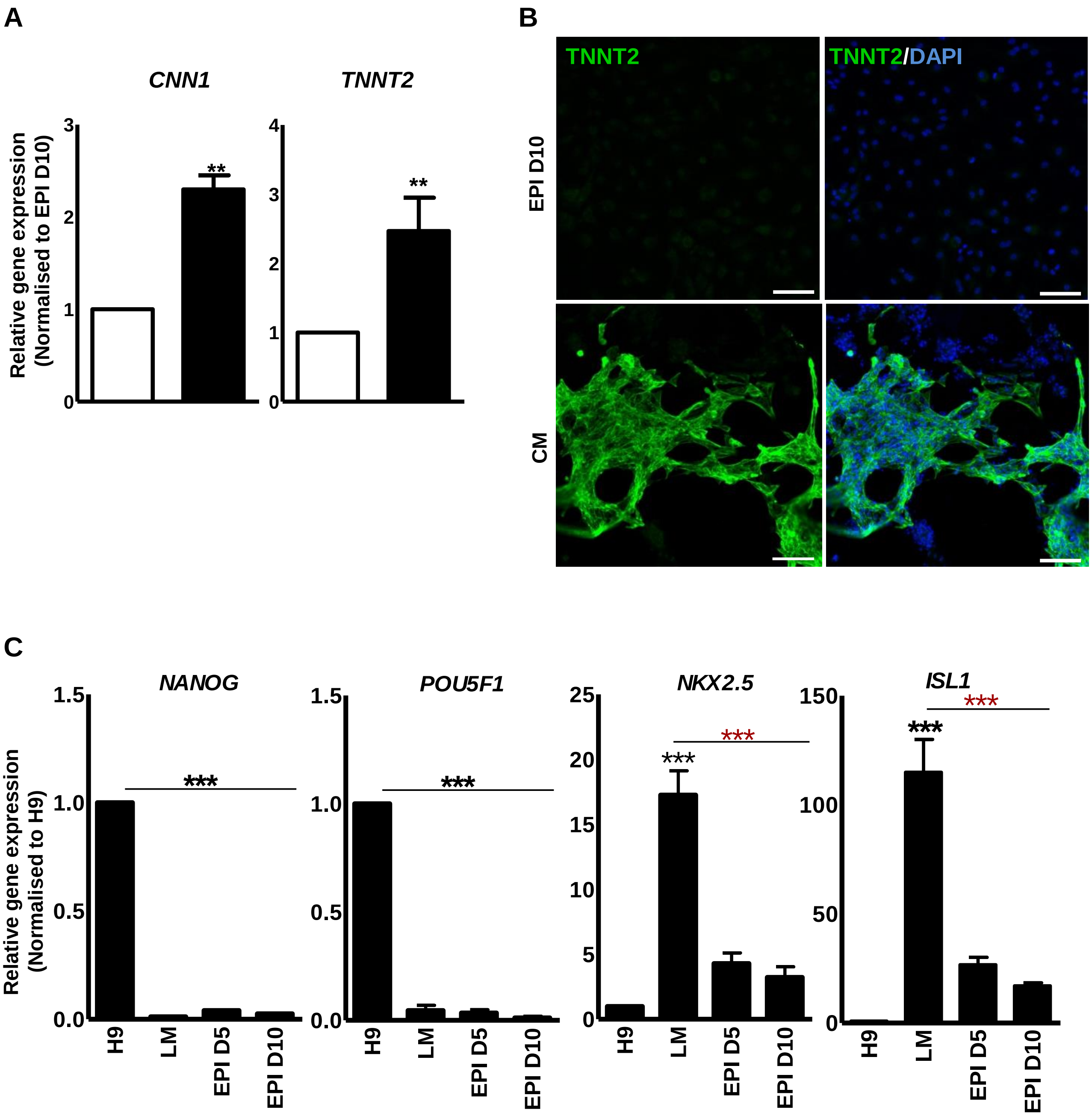

Fig. S3. HESC-derived epicardial cells express SMC and cardiomyocyte markers at longer periods of differentiation. (A) Analysis of SMC (*CNN1*) and cardiomyocyte (*TNNT2*) markers in the LM-derived day 10 (EPI D10) and day15 (EPI D15) epicardial cells by qRT-PCR. $^{***}P<0.001$, $^{**}P<0.01$. (B) Immunocytochemical analysis of TNNT2 in EPI D10 and H9-derived cardiomyocytes (CM). (C) Analysis of pluripotency (*NANOG*, and *POU5F1*) and LM (*NKX2.5* and *ISL1*) markers in H9, LM and epicardial cells after 5 (EPI D5) and 10 (EPI D10) days of differentiation. Significant differences compared to H9 are indicated in black, whereas differences between groups are indicated in red. $^{***}P<0.001$, $^{**}P<0.01$, $^{*}P<0.05$.

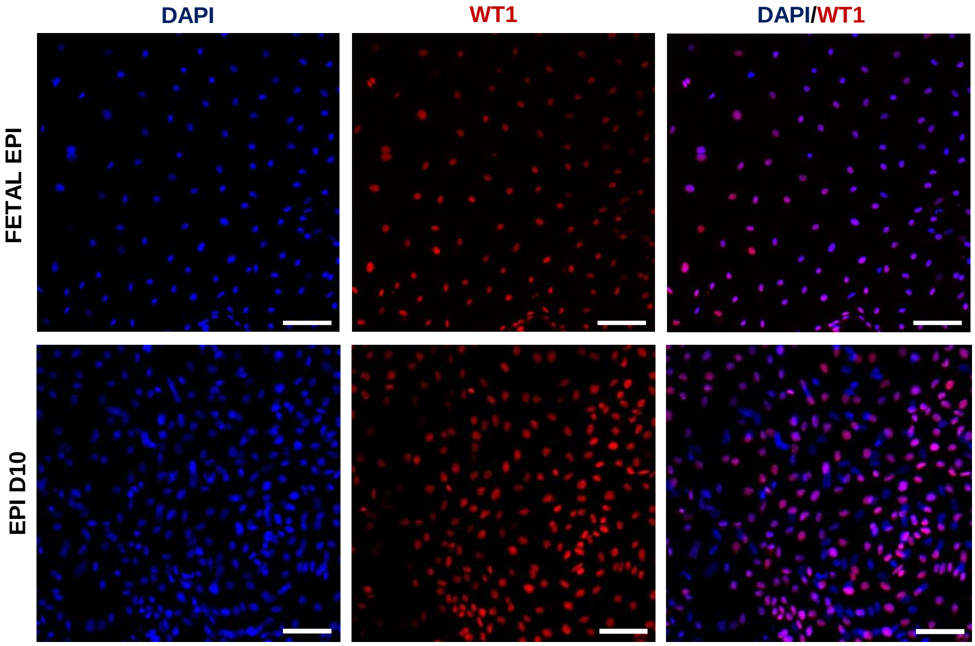

Fig. S4. HESC-derived epicardial cells are similar to human fetal epicardium. Human fetal epicardial outgrowths (FETAL EPI) and H9-derived epicardial cells (EPI D10) displayed similar WT1 expression. Scale bar, 100 μm.

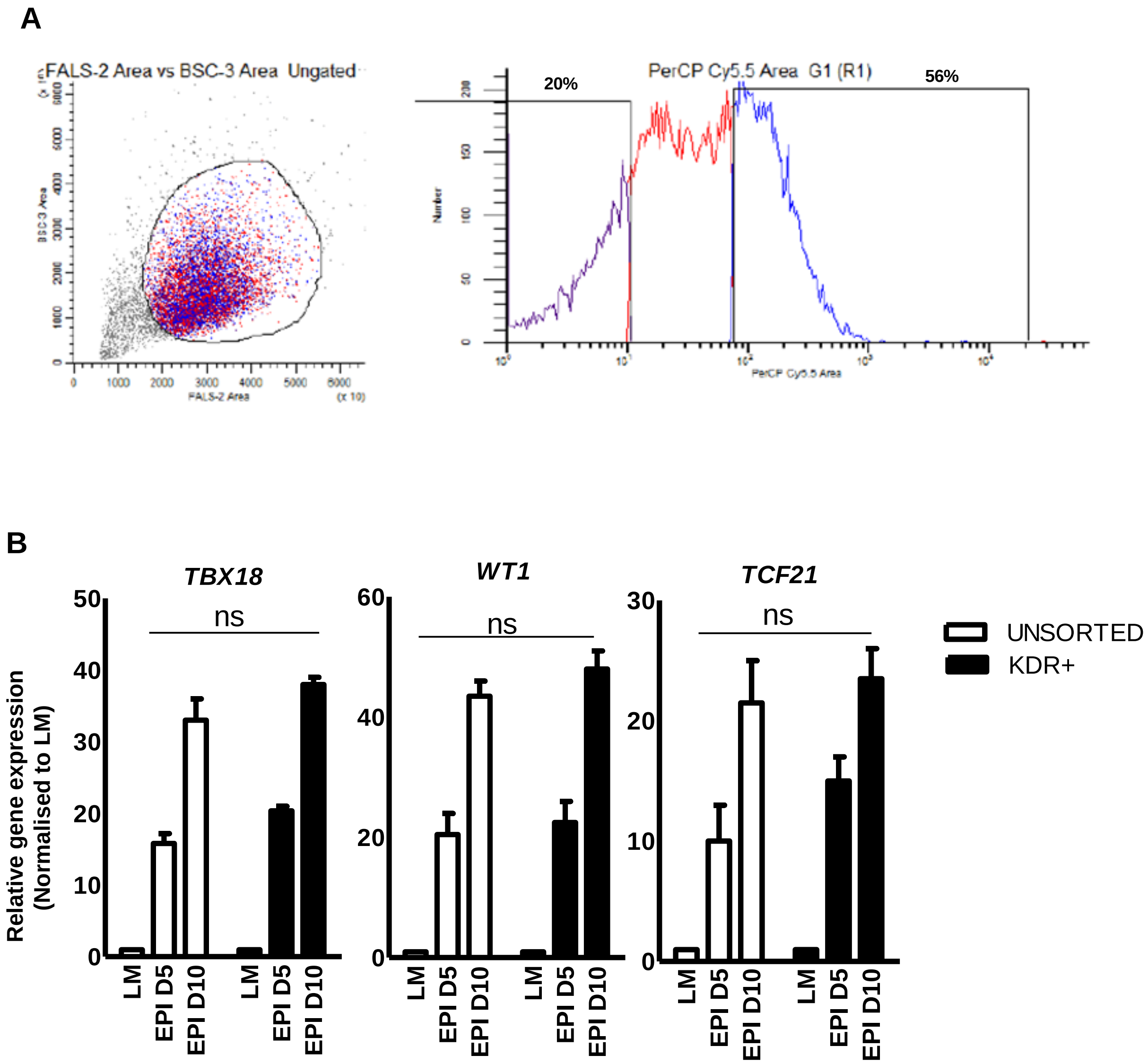

Fig. S5. Epicardium differentiation from flow sorted lateral plate mesoderm (LM). (A) Sort plot demonstrating 56% KDR^+ (blue) and 20% KDR^- (purple) LM cells. (B) Analysis of epicardial genes by qRT-PCR in epicardium-like cells derived from flow sorted and unsorted LM. Comparisons between the sorted and unsorted groups were made using Two-Way ANOVA.

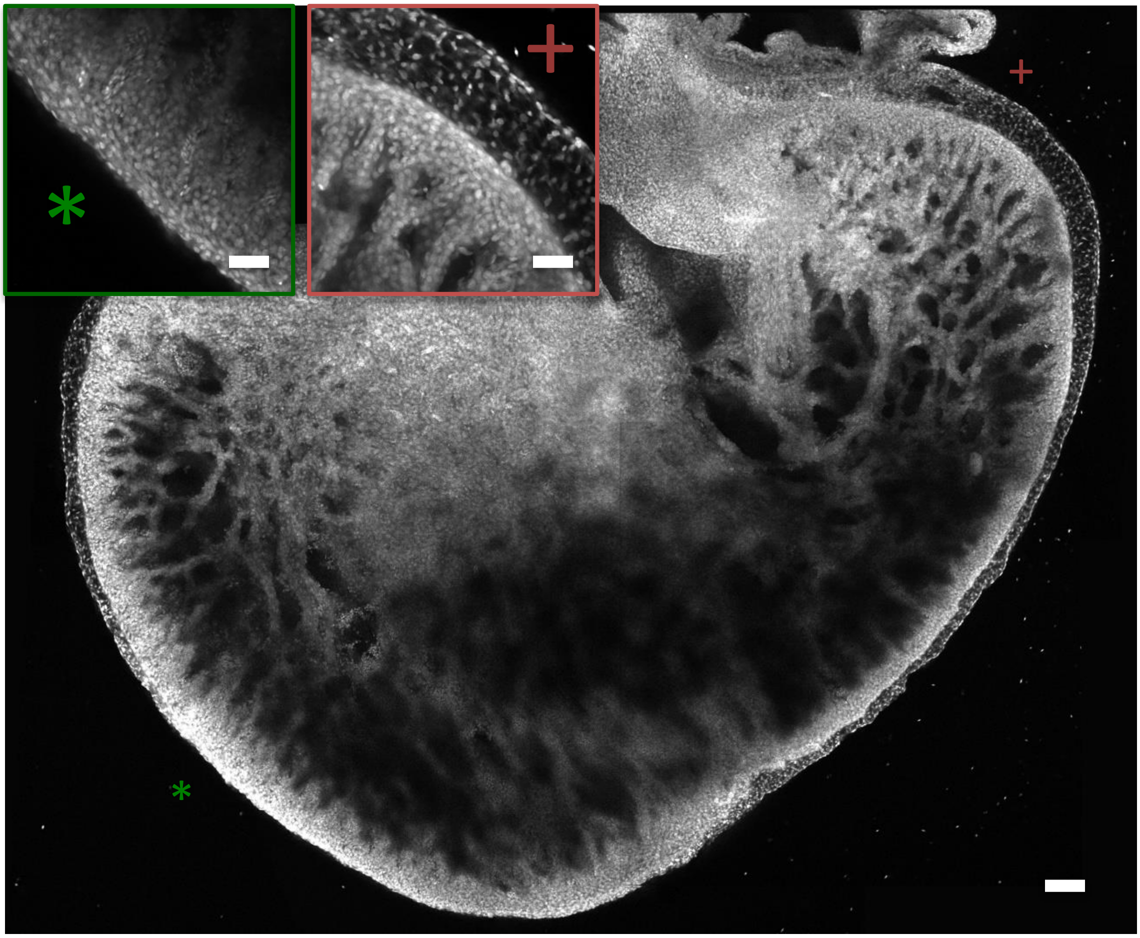

Fig. S6. Localisation of human epicardial cells in the subepicardial/epicardial region *in vivo*. A chicken heart at HH34 reconstructed from confocal sections. GFP^{+} or mStrawb^{+} epicardial cells were mostly seen in the subepicardial space between the epicardium and myocardium at the base of the heart (indicated by red +). WT1 expressing epicardial cells were spotted at the apex of the heart (indicated by green*) where the epicardial/subepicardial region was relatively thinner. The scale bar represents 285 mm in the large panel and 100 mm in the two small inserted panels.

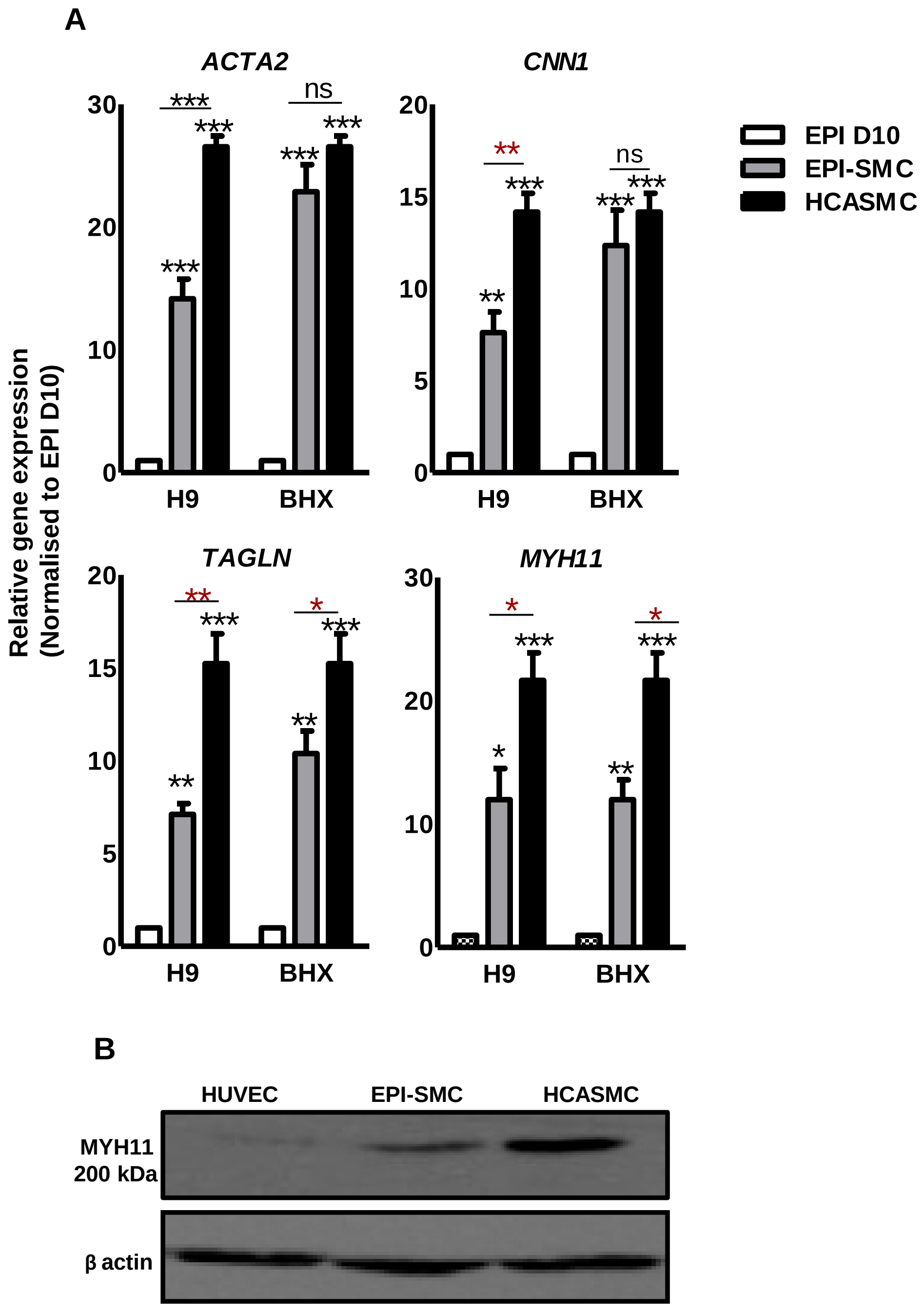

Fig. S7. HPSC-derived epicardial cells differentiate into mature vascular SMCs. (A) Analysis of SMC markers in epicardium-derived SMCs (EPI-SMC) generated from H9 HESCs and BHX HiPSCs and their comparison with human coronary artery SMCs (HCASMCs) by qRT-PCR. Significant differences compared to day 10 epicardium (EPI D10) are indicated in black, whereas differences between groups are indicated in red. $^{***}P<0.001$, $^{**}P<0.01$, $^{*}P<0.05$. (B) Western Blot analysis of mature SMC protein MYH11 in human umbilical vein endothelial cells (HUVECs), EPI-SMCs and HCASMCs. β-actin was used as loading control.

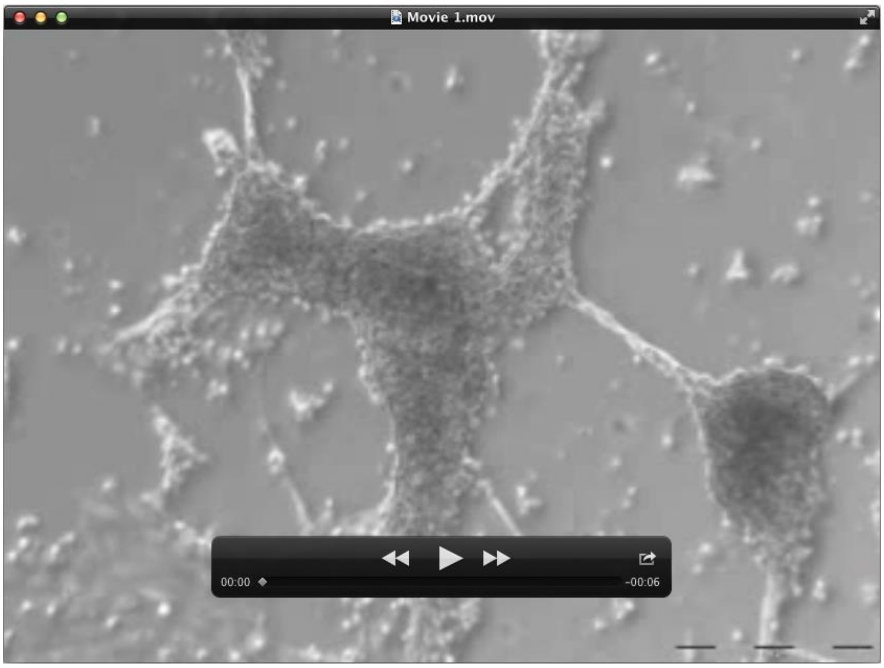

Movie 1 : This movie shows beating cardiomyocytes that were derived from human embryonic stem cells. These cells were used as a positive control in figure S3 to examine the expression of myocardial marker TNNT2.

Table S1

Gene	Sequence
NKX2.5	AGCCGAAAAGAAAGAGCTGTGCG<br>GACCTGCGCCTGCGAGAAGAG
ISL1	GCAAATGGCAGCGGAGCCCA<br>AGCAGGTCCGCAAGGTGTGC
FOXF1	GTACCCGCACCACGACAGCTC<br>ATACCGCGGGATGCCTTGCAG
PITX2	GCGTGTGTGCAATTAGGCG<br>AAAGTGAGTCCGCTGCCGCC
PBGD	GGAGCCATGTCTGGTAACGG<br>CCACGCGAATCACTCTCATCT
TBX18	ACTCCGGGCGCAACAGAATGG<br>TGGGCCCCAGATGGAAGGCA
WT1	TCCGGCCCAGTTTGTAGTAGA<br>AGCAGGGCTCGCTGGGTGAG
TCF21	GAAGTGGTGACCGCGAGCCG<br>AGTGTTCTCGCGGGGTGGGA
BNC1	GGGAACTGTGAAGGGTCGAG<br>TCGACTGCGAACAGACGAAA
UPK1B	AGGACAATTGCTGTGGCGTA<br>GGAGAACCCAAAAAGTCCAGC
ANXA8	ACACGAATCCATCCCAACCGAGAT<br>AACACAGTGTCCTTGGGTCAGGAA
ACTA2	CACTGTCAGGAATCCTGTGA<br>CAAAGCCGGCCTTACAGA
CNN1	GTCCACCCTCCTGGCTTT<br>AAACTTGTTGGTGCCCATCT
TAGLN	TCTTTGAAGGCAAAGACATGG<br>TTATGCTCCTGCGCTTTCTT
MYH11	AGATGGTTCTGAGGAGGAAACG<br>AAAACTGTAGAAAGTTGCTTATTCACT
CDH1	GGCTGGACCGAGAGAGTTTC<br>CGACGTTAGCCTCGTTCTCA

VIM	GAAGGCGAGGAGAGCAGGATT<br>CAAGGTCATCGTGATGCTGAG
OCLN	CGGCGAGCGGATTGGTT<br>TAGGCTGGCTGAGAGAGCATT
ZEB1	CATATTGAGCTGTTGCCGCTG<br>TCTTGCCCTTCCTTTCCTGTGT
NANOG	TCCTGAACCTCAGCTACAAACA<br>GGTAGGTGCTGAGGCCTTCT
POU5F1	GTGGGGGCAGGGGAGTTTGG<br>AGTGTGTCTATCTACTGTGTCCCAGGC
CD34	CACAGGAGAAAGGCTGGGCGA<br>TGGCCGTTTCTGGAGGTGGC
POSTN	CCCGTGACTGTCTATAAGCCAA<br>GTGTGTCTCCCTGAAGCAGT
DDR2	TTCTTTTTGGGTTGGGGAAACG<br>TGGGTCCTGGGAGGCATATCA
NOS3	AAGGCTTTTGATCCCCGGGTCCT<br>TCTCCATCAGGGCAGCTGCAAAG
PDGFRA	ATCGGAGGAGAAGTTTCCCAGAG<br>GGTACTGCCAGCTCACTTCA

Table S2

Marker	Application	Concentration	Manufacturer
ISL1	Flow cytometry<br>Immunocytochemistry	1:200<br>1:250	Abcam (ab26122)
KDR-PerCP	Flow cytometry	10µl added to 100µl of 10^6 cells	R&D Systems (FAB357C)
NKX2-5	Immunocytochemistry	1:200	Santa Cruz (SC-14033)
WT1	Flow cytometry<br>Immunocytochemistry<br>Western Blot	1:100<br>1:50<br>1:1000	Abcam (ab89901)
BNC1	Immunocytochemistry	1:50	Dr Shiro Luchi and Professor Howard Green
TCF21	Western Blot	1:250	Abcam (ab170395)
ACTA2-FITC	Flow cytometry<br>Immunocytochemistry	1:400<br>1:400	Sigma (F3777)
CNN1	Flow cytometry<br>Immunocytochemistry	1: 15000<br>1: 15000	Sigma (C2687)
TAGLN	Immunocytochemistry	1:1000	Abcam (ab14106)
ACTA2	Whole mount Immunofluorescence	1:200	Dako (M0851)
MYH11	Western blot	1:5000	Sigma (M7786)
DDR2	Immunocytochemistry	1:200	Santa Cruz (sc7555)
POSTN	Flow cytometry	1:500	Abcam (ab14041)

HOECHST 33342	Whole mount immunofluorescence	1:250	Sigma (14533)
β-ACTIN	Western blot	1:5000	Sigma (A1978)
